# Quality of Adult Inpatient Discharge Planning and 3 Day Follow Up – a Regional Audit

**DOI:** 10.1192/bjo.2024.561

**Published:** 2024-08-01

**Authors:** Tajnin Mitu, Anita Pierce, Nehad Seddiq, Asmaa Elsayed, Ishraq Elahi

**Affiliations:** 1 Betsi Cadwaladr University Health Board, Wrexham, United Kingdom; 2 Betsi Cadwaladr University Health Board, Rhyl, United Kingdom; 3 Betsi Cadwaladr University Health Board, Bangor, United Kingdom

## Abstract

**Aims:**

This study aimed to assess the post-discharge follow-up processes for psychiatric patients, specifically focusing on a 72-hour follow-up with documented Mental State Examination (MSE) and the presence of a comprehensive care plan, including up-to-date risk assessments and handover documentation.

**Methods:**

Conducted across three psychiatric units – Heddfan, Ablett, and Hergest – and associated Community Mental Health Team (CMHT) sites within Betsi Cadwaladr University Health Board, the audit spanned eight weeks (14/08/2023 to 16/10/2023). Adhering to NICE guidelines (NG-53) and CCQI Standards for Community-Based Mental Health Services-2017, data collection focused on the specified criteria.

**Results:**

Analysis revealed that 23% of patients did not receive a 72-hour follow-up post-discharge, attributed to reasons such as patient refusal or missed appointments. Only 74% of patients had documented risk assessments, posing challenges to follow-up teams. Despite the hospital's controlled environment, transitioning patients into the community demands updated risk assessments. While 87% of patients had documented mental state examinations during follow-ups, there's room for improvement in this crucial activity.

**Conclusion:**

In summary, the study emphasizes the importance of meticulous documentation and communication in the transition from inpatient psychiatric care to community settings. Challenges in achieving comprehensive follow-up documentation, with only 67% meeting criteria, were identified. The presence of an online Medication Therapy and Electronic Discharge system faced obstacles in printout availability. Designating a responsible individual for care plans pre-discharge and commendable adherence to thorough assessments during inpatient stays (83%) underscore efforts for a holistic approach. Future enhancements should target improving medication information integration and fortifying collaboration between inpatient and community teams. Addressing these aspects not only prevents medication-related errors but also ensures a seamless and patient-focused transition, enhancing the overall quality of mental health care delivery.

Additional Authors: Dr Minahil Junaid, Betsi Cadwaladr University Health Board, Rhyl; Dr Moheet Rahal, Betsi Cadwaladr University Health Board, Rhyl; Dr Aanika Nawr Hoque, Betsi Cadwaladr University Health Board, Wrexham; Raghdah Faisal, Betsi Cadwaladr University Health Board, Bangor, Amin Rezk, Betsi Cadwaladr University Health Board, Wrexham; Mostafa Negm, Betsi Cadwaladr University Health Board, Wrexham.

